# Enhancing Cryopreservation Efficiency in *Populus davidiana* × *P. tremuloides* Shoot Tips: Optimization of Vitrification Protocols and Mechanistic Insights into Flavonoid-Mediated Stress Adaptation

**DOI:** 10.3390/plants15010018

**Published:** 2025-12-20

**Authors:** Panke Yang, Zelin Li, Yu Qi, Yuandong Ma, Chunming Li, Maolan Liu, Wenjun Ma, Hui Bai, Huanzhen Liu

**Affiliations:** 1 College of Forestry, Northeast Forestry University, Harbin 150040, China; 15239653907@163.com (P.Y.); lzl0617521@163.com (Z.L.); 15244696259@163.com (Y.Q.); mayd1272@163.com (Y.M.); lichunming@nefu.edu.cn (C.L.); 15085310766@163.com (M.L.); 19811808592@163.com (W.M.); 2 Heilongjiang Provincial Forestry Research Institute, Harbin 150040, China

**Keywords:** *Populus davidiana* × *P. tremuloides*, cryopreservation, vitrification, flavonoid biosynthesis, oxidative stress, multi-omics analysis

## Abstract

Cryopreservation is vital for conserving the elite germplasm of the hybrid poplar *Populus davidiana* × *P. tremuloides*, which is difficult to propagate conventionally. This study established optimized vitrification and encapsulation–vitrification protocols, achieving high regeneration rates of 85.91% and 79.70%, respectively, with confirmed genetic stability. The process induced oxidative stress, altering markers (MDA, H_2_O_2_) and antioxidant enzyme activities (SOD, POD, CAT). Integrated transcriptomic and metabolomic analysis of key steps—preculture/loading (DLA) and osmotic dehydration (DLB)—revealed extensive stress-responsive reprogramming. A central finding was the robust activation of the flavonoid biosynthesis pathway during DLB, marked by upregulation of key genes (PAL, CHS) and accumulation of flavonols (e.g., quercetin). This response was linked to hormone signaling and antioxidant systems, forming a coordinated defense network. Our multi-omics findings demonstrate that successful cryopreservation relies on an adaptive response where flavonoid biosynthesis plays a critical role in conferring oxidative stress tolerance, providing a theoretical basis for improving woody plant cryopreservation.

## 1. Introduction

*Populus* spp. serve as economically valuable timber species and ecologically important protective trees. The sustainable preservation of their superior germplasm resources constitutes a fundamental prerequisite for the advancement of forestry [1]. The hybrid *Populus davidiana* × *P. tremuloides*, combining the genetic backgrounds of Chinese and American aspen, displays excellent adaptability and fast growth, indicating high breeding potential. Nevertheless, the commercial utilization and preservation of its superior genotypes are critically limited by the inherently low rooting efficiency during cutting-based vegetative propagation [2]. In this context, cryopreservation represents a pivotal strategy to overcome this bottleneck, enabling the long-term and stable conservation of germplasm resources.

In plant cryopreservation, vitrification-based methods—specifically the vitrification method and the encapsulation–vitrification method—have been established as key techniques for achieving high survival and regeneration rates [3]. The vitrification method involves pretreating plant shoot tips with a specific vitrification solution followed by rapid cooling to induce a vitrified state, thereby enabling long-term genetic storage [4]. The encapsulation–vitrification method combines vitrification with encapsulation; before loading, shoot tips are mixed with sodium alginate solution and embedded, after which the beads are permeated with vitrification solution prior to rapid freezing [5]. To improve permeation of cryoprotectants across the cell membrane and to enhance tolerance to the dehydration stress imposed by vitrification solutions, a pretreatment step known as “loading” is commonly applied [6]. This process reduces tissue water content, mitigates injury from abrupt osmotic shifts, and increases intracellular levels of protective compounds [7]. In some cases, however, loading alone is insufficient to confer full tolerance, making preculture necessary before proceeding [8]. Osmotic dehydration represents the critical phase for achieving glass transition [9]. By using high-concentration vitrification solutions such as PVS3, the cytoplasm is further concentrated to a critical threshold, markedly raising intracellular viscosity and suppressing ice nucleation. This allows cells to form a stable glassy state rather than lethal ice crystals during freezing [10]. Finally, ultra-rapid freezing in liquid nitrogen instantly solidifies the dehydrated cytoplasm into an amorphous glass [11], thereby completely circumventing the structural damage caused by ice crystallization. Thus, the efficacy of both cryopreservation techniques relies on three tightly linked core steps: preculture/loading, osmotic dehydration, and liquid-nitrogen freezing [12].

Although the vitrification technique is well-established, its crucial pretreatment phase—spanning from loading with low-concentration protectants to exposure to the high-concentration PVS2 solution—concurrently primes the tissue for vitrification yet progressively subjects it to physiological stress [13]. The preculture/loading step, conducted in a low-toxicity environment to equilibrate osmotic pressure, induces mild water and preliminary oxidative stresses, thereby priming the material for subsequent PVS2 exposure [14]. The PVS2 treatment itself induces severe cellular dehydration via its hypertonic environment [15], while its chemical constituents directly disrupt metabolic homeostasis. This synergy disrupts reactive oxygen species (ROS) metabolism, leading to oxidative damage manifestations such as membrane lipid peroxidation, protein denaturation, and nucleic acid impairment [16].

To mitigate the aforementioned stresses, plant cells maintain intracellular redox homeostasis through an orchestrated coordination of enzymatic antioxidants [17] (e.g., SOD, CAT, POD, APX) and non-enzymatic antioxidants [18] (e.g., GSH, AsA) [19]. The efficacy of this integrated antioxidant system is a decisive factor for cellular viability under such stressful conditions [20]. Consequently, the physiological state and survival potential of cells prior to cryogenic exposure are fundamentally governed by their comprehensive resilience and adaptive capacity to the multi-step pretreatment regimen [21]. Presently, however, a systematic analysis is lacking regarding how the antioxidant system regulates the dynamic equilibrium between cumulative damage and stress adaptation throughout the continuous loading-to-osmotic treatment process.

Therefore, using shoot tips of *Populus davidiana* × *P. tremuloides* as experimental material in a vitrification-based cryopreservation system, this study systematically compared the survival and regeneration performance of materials after each procedural step, along with conducting related physiological and biochemical assays. Transcriptomic and metabolomic analyses were conducted on three critical stages—preculture/loading (DLA), osmotic dehydration (DLB), and liquid nitrogen freezing (DLC)—to comprehensively elucidate the dynamic interaction networks underlying cellular damage and protection, spanning from stress acclimation through deep freezing. The findings will not only clarify the mechanisms of stress-induced cross-tolerance during early procedural stages but also provide theoretical support for optimizing poplar germplasm cryopreservation protocols, while advancing the understanding of plant adaptive responses under combined-stress conditions.

## 2. Results

### 2.1. Effect of Cold Acclimation Duration on Shoot Tip Cryopreservation in Populus davidiana × P. tremuloides

**Figure 1:**
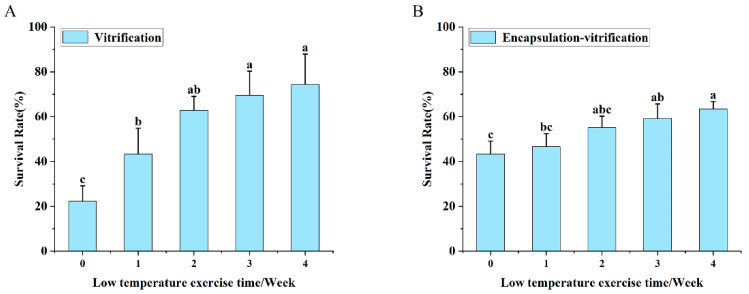
Cryopreservation survival of shoot tips in response to varying cold acclimation periods. (**A**) Vitrification. (**B**) Encapsulation–vitrification. Values labeled with different lowercase letters are significantly different (*p* < 0.05).

Under the vitrification (Figure 1A) and encapsulation–vitrification (Figure 1B) cryopreservation methods, the survival rate of shoot tips exhibited a significant increase with prolonged cold acclimation duration. The highest survival rates, reaching 74.44% and 63.43%, respectively, were achieved after a 4-week acclimation period.

### 2.2. Effect of Preculture on Shoot Tip Survival Following Cryopreservation

**Figure 2:**
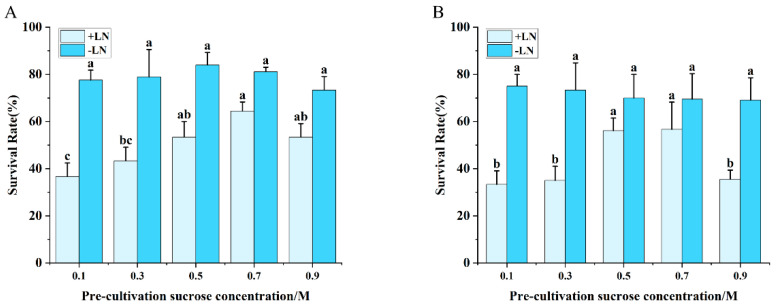
Effect of preculture sucrose concentration on shoot tip survival after cryopreservation. (**A**) Vitrification method. (**B**) Encapsulation–vitrification method. Values labeled with different lowercase letters are significantly different (*p* < 0.05).

In both cryopreservation methods, shoot tip survival exhibited a concentration-dependent increase with elevated sucrose levels in the preculture medium. The vitrification method (Figure 2A) attained a maximal survival rate of 64.00% at 0.7 mol/L sucrose, whereas the encapsulation–vitrification method (Figure 2B) achieved its peak survival of 56.11% at 0.5 mol/L.

**Figure 3:**
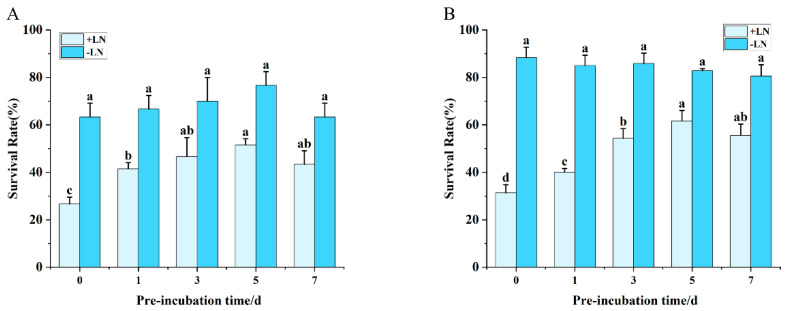
Effect of preculture duration on shoot tip survival following cryopreservation. (**A**) Vitrification method. (**B**) Encapsulation–vitrification method. Values labeled with different lowercase letters are significantly different (*p* < 0.05).

The survival rate of shoot tips during preculture exhibited a distinct bell-shaped response over time (Figure 3). For both cryopreservation techniques, survival peaked on the 5th day of preculture, reaching 51.52% with the vitrification method (Figure 3A) and 61.67% with the encapsulation–vitrification method (Figure 3B), after which a progressive decline was observed. No significant differences were detected among the parallel controls (−LN) across treatments.

This pattern can be attributed to the dual role of preculture. While optimal conditions enhance desiccation tolerance, prolonged exposure or excessive sucrose concentrations exacerbate osmotic stress, becoming detrimental to survival. Therefore, identifying the critical balance in sucrose concentration and preculture duration is essential to moderately reduce tissue water content, bolster cryotolerance, and ultimately maximize post-thaw regeneration.

### 2.3. Effect of Vitrification Solution and Exposure Duration on Shoot Tip Survival During Cryopreservation

**Figure 4:**
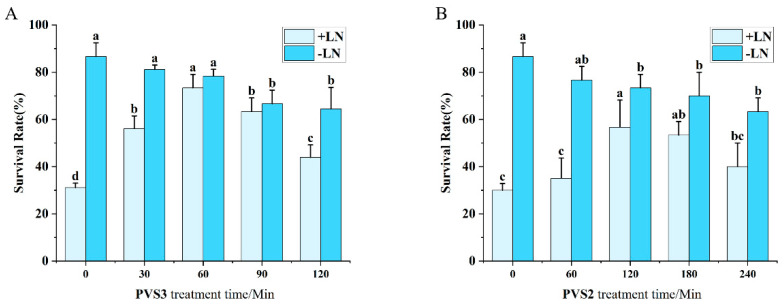
Effect of vitrification solution exposure duration on shoot tip survival following cryopreservation. (**A**) Vitrification method. (**B**) Encapsulation–vitrification method. Values labeled with different lowercase letters are significantly different (*p* < 0.05).

Exposure to vitrification solutions is a critical protective step prior to cryopreservation, serving to reduce intracellular free water content. In this study, PVS2 and PVS3 were evaluated for their effects on shoot tip survival. Under the vitrification protocol (Figure 4A), survival exhibited a biphasic effect in response to PVS3 exposure time, peaking at 73.33% after 60 min before declining. In contrast, the encapsulation–vitrification method (Figure 4B) achieved optimal survival (56.67%) following 120 min of PVS2 treatment. In non-frozen controls (–LN), prolonged exposure to both solutions progressively reduced survival, consistent with cumulative osmotic and chemical injury. Notably, PVS2 induced a more pronounced toxicity compared to PVS3, as reflected by the steeper decline in control survival over time.

### 2.4. Establishment of a Shoot Tip Cryopreservation System for Populus davidiana × P. tremuloides

**Figure 5:**
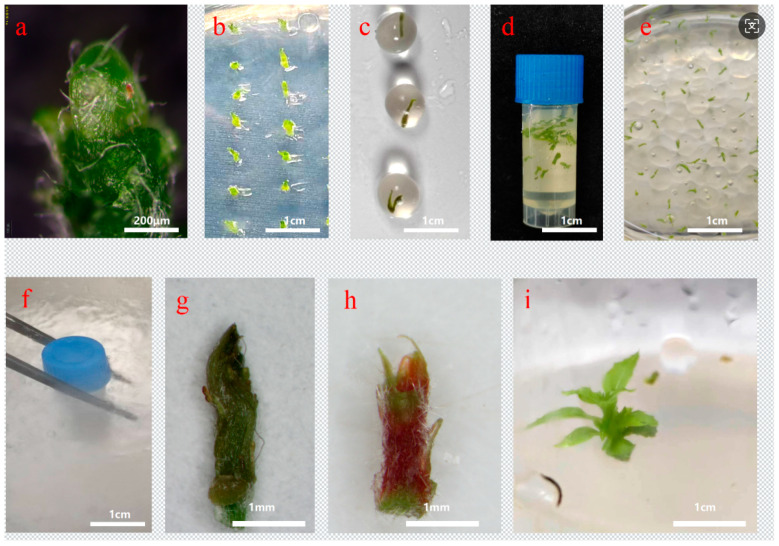
Cryopreservation process and regeneration of *Populus davidiana* × *P. tremuloides* shoot tips. (**a**) Shoot tip explant. (**b**) Preculture on sucrose-enriched medium. (**c**) Encapsulation in alginate beads. (**d**) Loading and PVS3 treatment. (**e**) Loading and PVS2 treatment of encapsulated shoot tips. (**f**) Liquid nitrogen storage. (**g**) Non-viable shoot tip stained with TTC. (**h**) Viable shoot tip stained with TTC. (**i**) Regenerated plantlet.

Based on the optimized parameters established in the preceding experiments, standardized protocols for the cryopreservation of in vitro shoot tips of *Populus davidiana* × *P. tremuloides* were developed (Figure 5).

Vitrification-based cryopreservation: Shoot tips (2 mm in length) excised from 4-week cold-acclimated plantlets (Figure 5a) were precultured in 0.7 mol/L sucrose medium at 4 °C for 5 days in darkness (Figure 5b). Subsequently, samples were subjected to loading treatment at room temperature for 20 min, followed by dehydration with PVS3 for 60 min (Figure 5d), and then plunged into liquid nitrogen for 1 h (Figure 5f). For rewarming, samples were immersed in a 40 °C water bath for 1 min, drained, and rinsed with recovery medium for 20 min. Tissues were then incubated in darkness for 7 days before transfer to standard light conditions.

Encapsulation–vitrification cryopreservation: Shoot tips precultured in 0.5 mol/L sucrose medium at 4 °C for 5 days in darkness were encapsulated in alginate/CaCl_2_ beads (Figure 5c). After loading for 20 min, samples were treated with PVS2 on ice for 120 min (Figure 5e), transferred to liquid nitrogen for 1 h (Figure 5f), rewarmed in a 40 °C water bath for 3 min, and rinsed with loading solution for 20 min before being returned to culture conditions identical to those used in the vitrification protocol.

Viable shoot tips exhibited dark pink pigmentation (Figure 5h), whereas non-viable ones turned brown (Figure 5g). After 4 weeks of culture, regenerated plantlets were obtained (Figure 5i). The optimized vitrification and encapsulation–vitrification protocols yielded regeneration rates of 85.91% and 79.70%, respectively.

### 2.5. Histological Analysis of Cell Survival in Cryopreserved Populus davidiana × P. tremuloides Shoot Tips

**Figure 6:**
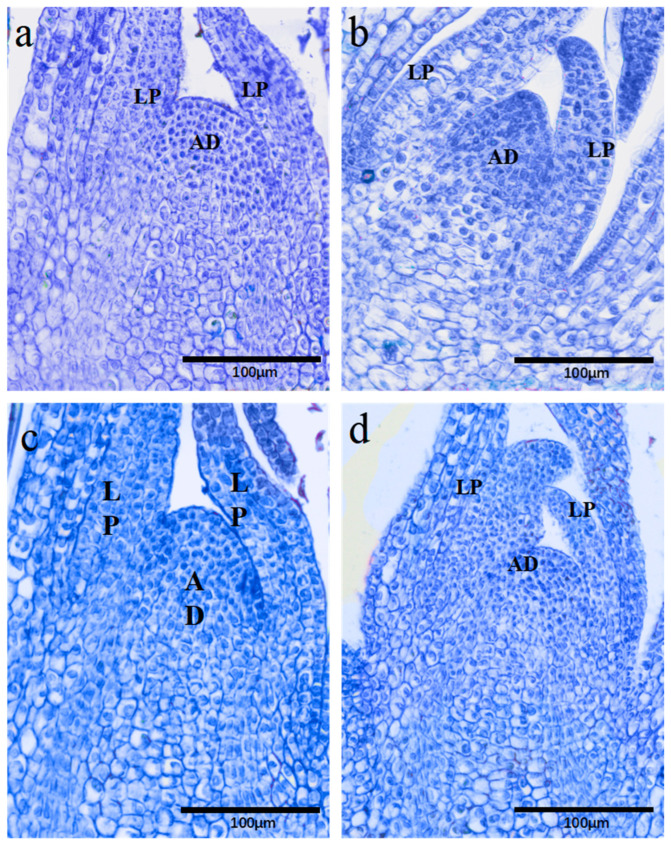
Histological analysis of shoot tip meristem survival following cryopreservation. (**a**) Positive control. (**b**) Negative control (fresh shoot tip directly immersed in liquid nitrogen). (**c**) Shoot tip processed by the vitrification method. (**d**) Shoot tip processed by the encapsulation–vitrification method. AD: apical meristem; LP: leaf primordium.

Histological analysis of cryopreserved shoot tips revealed that surviving cells were predominantly located in the apical meristem (layers 1–6) and the primordia of the first three leaves. These cells exhibited intensely stained cytoplasm and well-preserved cellular integrity (Figure 6a). In contrast, non-frozen control shoot tips directly immersed in liquid nitrogen (negative control) displayed lightly stained cells with ruptured membranes and severe structural disruption (Figure 6b). Shoot tips processed with both cryopreservation methods showed cytological features comparable to those of viable cells, including densely stained cytoplasm, intact membranes, and preserved nuclear structure, with no evident cryo-damage (Figure 6c,d).

### 2.6. Assessment of Genetic Stability in Regenerated Plants Following Cryopreservation

**Figure 7:**
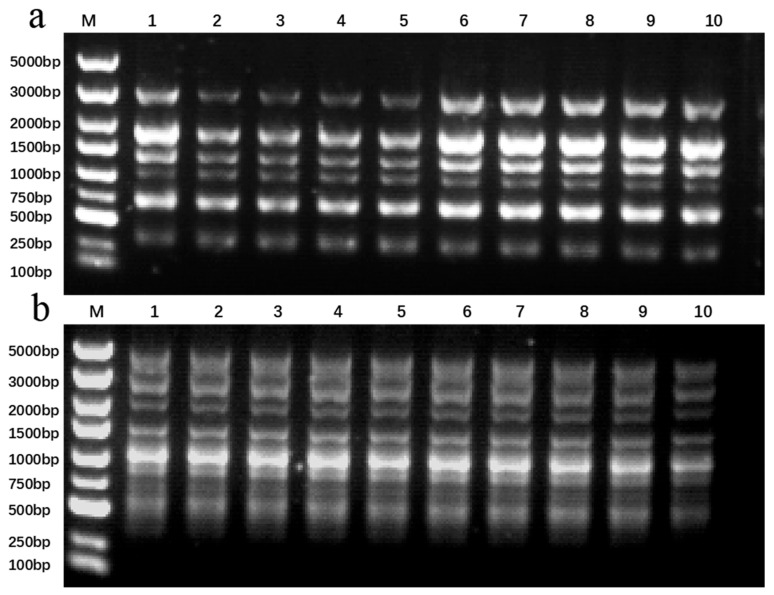
ISSR analysis of *Populus davidiana* × *P. tremuloides* plantlets regenerated after cryopreservation. M: DL5000 DNA marker; Lane 1: non-cryopreserved control plantlet; Lanes 2–10: regenerated plantlets following cryopreservation. (**a**) Profiles generated using primer UBC874 following vitrification. (**b**) Profiles generated using primer UBC888 following encapsulation–vitrification.

Plantlets regenerated following cryopreservation exhibited comparable performance to the non-cryopreserved controls in terms of rooting and vegetative growth (Table 1), indicating no phenotypic alterations induced by the cryopreservation process. Genetic stability was further confirmed by ISSR analysis, in which eight primers generated 50 reproducible bands (4–9 bands per primer; mean: 6.25). Both cryopreservation methods produced monomorphic banding patterns identical to the control across all samples (Figure 7; Table 2), demonstrating the preservation of genetic integrity in *Populus davidiana* × *P. tremuloides* after cryopreservation.

### 2.7. Physiological and Biochemical Analysis of the Cryopreservation Process of Stem Tips of Populus davidiana × P. tremuloides

**Figure 8:**
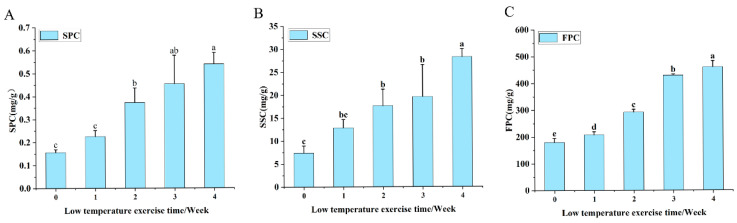
Effects of cold acclimation duration on osmo-protectant contents in *Populus davidiana* × *P. tremuloides*. SPC, soluble protein content; SSC, soluble sugar content; FPC, free proline content. Values labeled with different lowercase letters are significantly different (*p* < 0.05). Panels (**A**–**C**) show the effects of cold acclimation duration on the soluble protein content, soluble sugar content, and free proline content of *Populus davidiana × P. tremuloides*, respectively.

During the cold acclimation period, the contents of soluble proteins, soluble sugars, and free proline in *Populus davidiana* × *P. tremuloides* progressively increased with prolonged treatment duration (Figure 8). Relative to non-acclimated controls, the levels of these osmolytes showed respective increases of 70.37% (Figure 8A), 73.82% (Figure 8B), and 60.95% (Figure 8C). The accumulation of these osmoregulatory substances is associated with enhanced tolerance to low-temperature stress, thereby contributing to improved shoot tip survival following cryopreservation.

**Figure 9:**
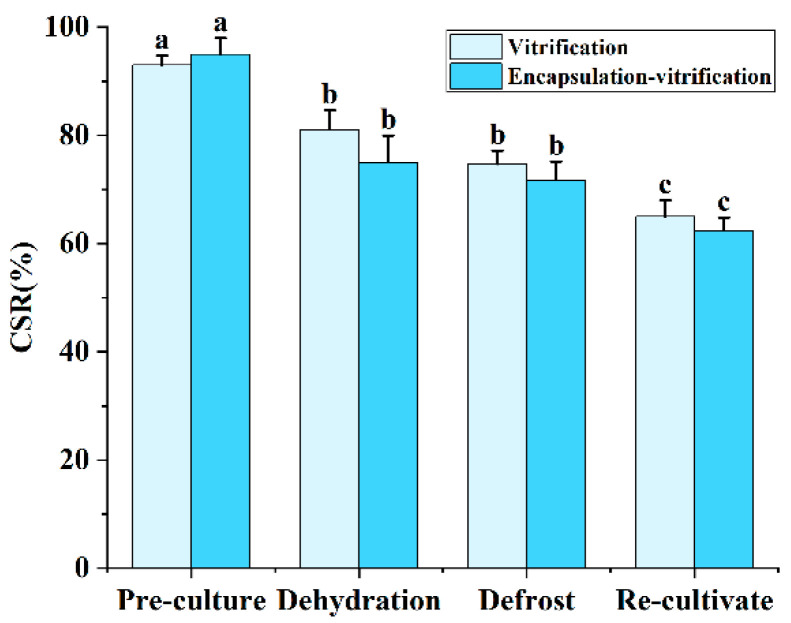
Changes in shoot tip cell viability following key steps of the cryopreservation process. CSR is cell survival rate. Values labeled with different lowercase letters are significantly different (*p* < 0.05).

Longitudinal assessment of shoot tip cell viability throughout the cryopreservation process is summarized in Figure 9. Initial pretreatment resulted in high cell survival rates (93–95%) without evident injury. Following dehydration, viability declined to 81% in the vitrification group and 75% in the encapsulation–vitrification group. This divergence is attributable to the composition of PVS3, which contains only sucrose and glycerol without DMSO, thus exerting relatively lower cytotoxicity. A further decrease in survival was observed after liquid nitrogen exposure, though not significantly different from dehydration levels. After recovery culture, final survival rates reached 65% for vitrification and 62% for encapsulation–vitrification.

**Figure 10:**
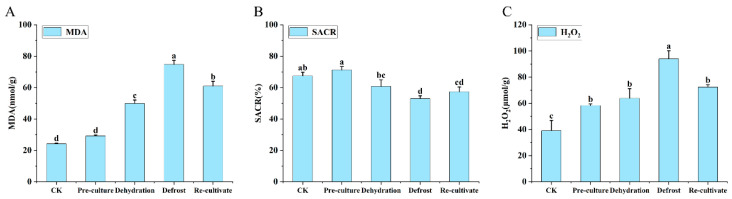
Changes in oxidative stress parameters during cryopreservation of *Populus davidiana* × *P. tremuloides* shoot tips. MDA, malondialdehyde; SACR, superoxide anion scavenging rate; H_2_O_2_, hydrogen peroxide. Values labeled with different lowercase letters are significantly different (*p* < 0.05). Panels (**A**–**C**) show the differences in MDA, SACR, and H_2_O_2_ levels during the cryopreservation process, respectively.

Throughout the cryopreservation process (Figure 10), malondialdehyde (MDA) concentration exhibited an initial increase followed by a decline, with all treatment groups maintaining levels significantly higher than those of the control (Figure 10A). The superoxide anion scavenging rate reached its maximum after preculture, decreased markedly during dehydration and thawing, and subsequently recovered gradually throughout the re-culture period (Figure 10B). Hydrogen peroxide (H_2_O_2_) concentration showed a pattern of rapid increase followed by a sharp decrease, with pronounced fluctuations observed specifically during the dehydration and thawing stages (Figure 10C).

**Figure 11:**
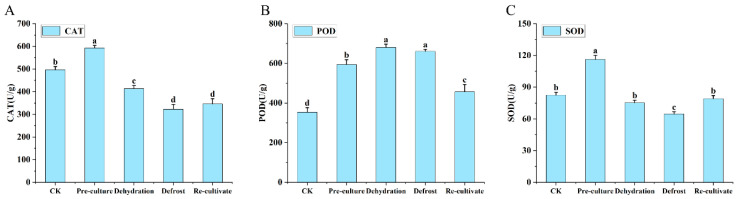
Changes in antioxidant enzyme activities during cryopreservation of *Populus davidiana* × *P. tremuloides* shoot tips. CAT, catalase; POD, peroxidase; SOD, superoxide dismutase. Values labeled with different lowercase letters are significantly different (*p* < 0.05). Panels (**A**–**C**) show the differences in CAT, POD, and SOD levels during the cryopreservation process, respectively.

Throughout the cryopreservation process (Figure 11), the three antioxidant enzymes exhibited divergent response patterns. CAT activity increased by 19.4% after pretreatment, declined to its nadir during thawing, and subsequently recovered slightly (Figure 11A). POD activity peaked following dehydration but decreased to 456 U/g after thawing, suggesting its role in early stress response and subsequent limitation due to cellular injury (Figure 11B). SOD activity displayed an overall decline initially, reaching the lowest level at thawing, followed by gradual recovery during re-culture (Figure 11C).

**Figure 12:**
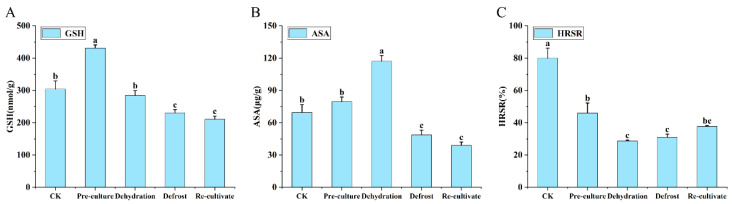
Changes in non-enzymatic antioxidants and hydroxyl radical scavenging capacity during cryopreservation of *Populus davidiana* × *P. tremuloides* shoot tips. GSH, reduced glutathione; ASA, ascorbic acid; HRSR, hydroxyl radical scavenging rate. Values labeled with different lowercase letters are significantly different (*p* < 0.05). Panels (**A**–**C**) show the differences in GSH, ASA, and HRSR levels during the cryopreservation process, respectively.

During cryopreservation (Figure 12), both reduced glutathione (GSH) and ascorbic acid (ASA) exhibited a biphasic pattern, characterized by an initial increase followed by a subsequent decrease. GSH concentration peaked during the preculture stage and progressively declined as the protocol advanced (Figure 12A). Similarly, ASA reached its maximum level after preculture, followed by a gradual reduction (Figure 12B). In contrast, the hydroxyl radical scavenging rate was highest in the control group, decreased significantly after preculture, reached its nadir during dehydration and thawing, and rebounded gradually upon re-culture (Figure 12C).

### 2.8. Correlation Analysis of Various Physiological Indicators During Cryopreservation

**Figure 13:**
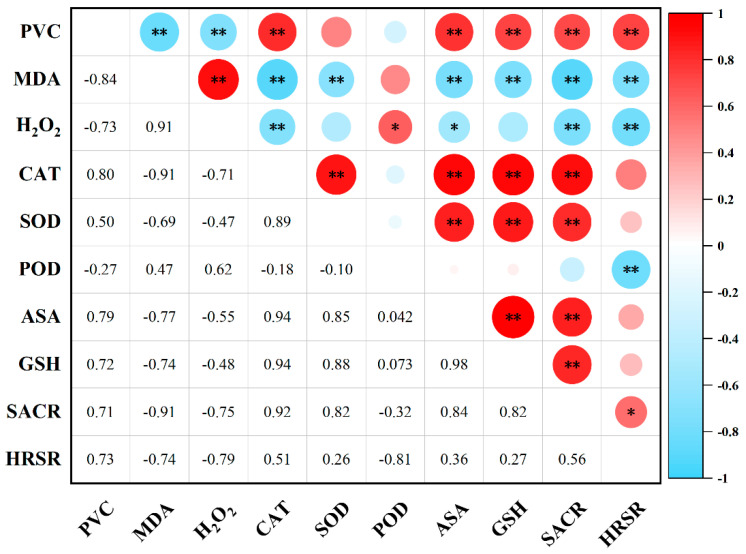
Interrelationships among physiological indicators during cryopreservation of *Populus davidiana* × *P. tremuloides* shoot tips. PVC, post-thaw viability of cells; SACR, superoxide anion scavenging rate; HRSR, hydroxyl radical scavenging rate. * indicates a significant difference (*p* < 0.05); ** indicates a highly significant difference (*p* < 0.01).

A significant dynamic correlation between oxidative damage and antioxidant defense was observed in *Populus davidiana* × *P. tremuloides* shoot tip cells during cryopreservation (Figure 13). Malondialdehyde (MDA) content showed significant negative correlations with catalase (CAT) and superoxide dismutase (SOD) activities, as well as with ascorbic acid (ASA) and reduced glutathione (GSH) levels (*p* < 0.01), indicating that severe oxidative damage suppresses antioxidant capacity. Hydrogen peroxide (H_2_O_2_) content was positively correlated with certain antioxidant components, potentially reflecting its role as a signaling molecule in early stress response activation. Strong positive correlation was observed between CAT and SOD activities (*p* < 0.01), demonstrating their synergistic action in reactive oxygen species elimination. Furthermore, ASA and GSH contents showed coordinated trends and were positively correlated with both superoxide anion and hydroxyl radical scavenging rates, highlighting the crucial role of non-enzymatic antioxidants in oxidative stress mitigation. Most importantly, post-thaw survival rate exhibited strong positive correlations with key antioxidant indicators including CAT, SOD, ASA, and GSH (*p* < 0.01), confirming that enhanced antioxidant defense capacity represents a key mechanism for improving cryopreservation efficiency.

### 2.9. Transcriptomics Analysis

#### 2.9.1. Screening of Differentially Expressed Genes

**Figure 14:**
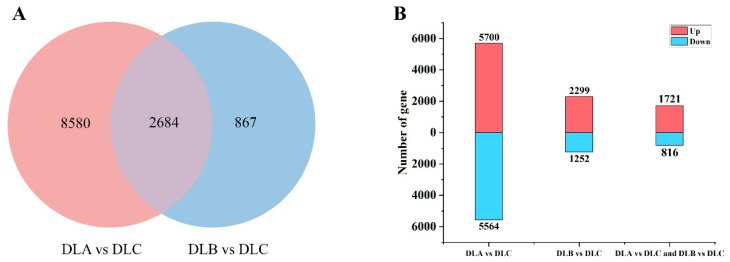
Identification and distribution of differentially expressed genes (DEGs). (**A**) Venn diagram illustrating overlapping and unique DEGs across different treatment steps. (**B**) Numbers of up- and down-regulated DEGs in paired comparisons. DLA, preculture/loading; DLB, osmotic dehydration; DLC, liquid nitrogen freezing (control).

Transcriptome analysis was performed on shoot tips of *Populus davidiana* × *P. tremuloides* subjected to three critical cryopreservation steps: preculture/loading (DLA), osmotic dehydration (DLB), and liquid nitrogen freezing (DLC) (Figure 14A,B). Under the threshold of|log_2_Fold Change| > 1 and PADJ < 0.05, 2684 common differentially expressed genes (DEGs) were identified. Considerable numbers of unique DEGs were detected among treatment groups, with the highest number specific to the DLC group, suggesting that osmotic dehydration exerted the most pronounced effect on transcriptional regulation. Statistical analysis of DEG numbers in paired comparisons (DLA vs. DLC, DLB vs. DLC) revealed a higher proportion of upregulated genes in the DLB vs. DLC group (Figure 14B), implying that osmotic dehydration may activate multiple stress-responsive pathways. In contrast, the DLC group, representing the liquid nitrogen freezing step, displayed relatively moderate transcriptomic alterations, indicating limited additional impact of freezing itself on gene expression. Both DLA and DLB treatments likely elicited extensive transcriptional reprogramming, presumably through physical stresses such as dehydration and osmotic perturbation. Among the identified DEGs, a substantial number are hypothesized to be associated with water stress, oxidative response, and cell wall remodeling, reflecting the adaptive mechanisms of *Populus davidiana* × *P. tremuloides* shoot tips during vitrification-based cryopreservation.

#### 2.9.2. GO and KEGG Enrichment Analysis of Differentially Expressed Genes (DEGs)

##### GO Enrichment Analysis of DEGs

**Figure 15:**
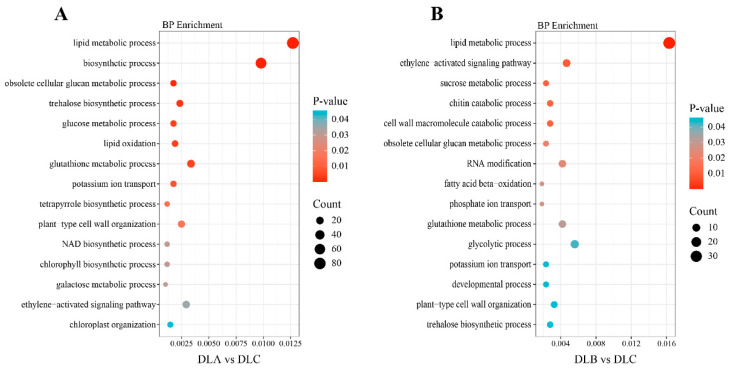
Gene Ontology (GO) Enrichment Analysis of Differentially Expressed Genes (DEGs). (**A**) GO Enrichment Analysis of DLA and DLC. (**B**) GO Enrichment Analysis of DLB and DLC. DLA, preculture/loading; DLB, osmotic dehydration; DLC, liquid nitrogen freezing (control).

To elucidate the functional impacts of different treatments on shoot tip development in *Populus davidiana* × *P. tremuloides*, Gene Ontology (GO) enrichment analysis was performed on key differentially expressed genes (DEGs) from DLA vs. DLC and DLB vs. DLC comparisons. Enrichment covered three major GO categories—biological process (BP), cellular component (CC), and molecular function (MF)—with emphasis on BP terms. Significantly enriched terms were visualized via bubble plot (Figure 15).

DEGs from both groups were significantly enriched in metabolic, stress-responsive, and cellular organization processes. DLA predominantly activated osmotic regulation, energy metabolism, and antioxidant defense mechanisms. Enriched processes such as trehalose biosynthesis and glutathione metabolism align with plant strategies for accumulating compatible solutes and scavenging reactive oxygen species under osmotic stress. Activation of potassium ion transport and cell wall organization reflects requirements for ionic homeostasis and structural remodeling. Furthermore, enrichment in chlorophyll and tetrapyrrole biosynthesis suggests that loading influenced photosynthetic metabolism, potentially as an adaptive preculture response prior to subsequent stress.

Compared to DLA, DLB significantly enriched catabolic and cell wall remodeling processes, including chitin and cell wall macromolecule decomposition, indicative of cell wall degradation and restructuring under severe dehydration. Enrichment of fatty acid β-oxidation and glycolysis revealed a shift toward energy-generating catabolism to support stress responses. Concurrently, persistent enrichment in phosphate and potassium ion transport underscored the importance of ion homeostasis in dehydrating environments.

Notably, both comparisons showed enrichment in ethylene signaling and glutathione metabolism, identifying these as core mechanisms in stress adaptation. Enrichment of developmental processes further implied long-term effects of dehydration on growth and differentiation, consistent with plants modulating developmental programs under stress.

##### KEGG Pathway Enrichment Analysis of Differentially Expressed Genes

**Figure 16:**
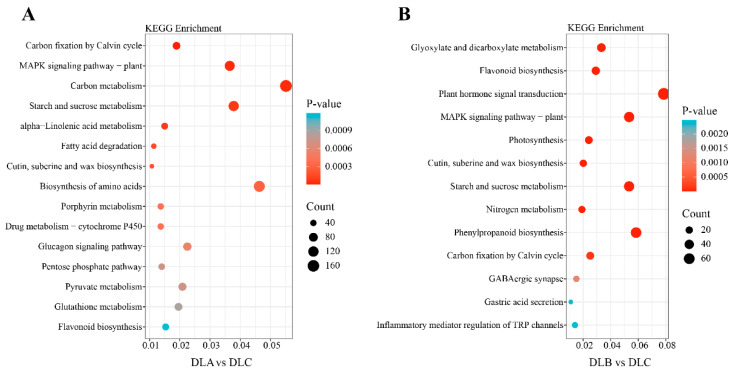
KEGG Pathway Enrichment Analysis of Differentially Expressed Genes (DEGs). (**A**) KEGG Pathway Enrichment Analysis of DLA and DLC. (**B**) KEGG Pathway Enrichment Analysis of DLB and DLC. DLA, preculture/loading; DLB, osmotic dehydration; DLC, liquid nitrogen freezing (control).

To systematically elucidate the biological pathways underlying transcriptomic differences between DLA vs. DLC and DLB vs. DLC, KEGG pathway enrichment analysis was performed on the differentially expressed genes. The results revealed significant enrichment of “Plant hormone signal transduction”, “Phenylpropanoid biosynthesis”, and “Starch and sucrose metabolism” pathways in both comparison groups (Figure 16). In terms of signaling and stress response, enrichment of the “MAPK signaling pathway-plant” and drug metabolism-cytochrome P450 pathways indicated that DLA may represent a physiological state involving active responses to environmental cues.

In the DLA vs. DLC comparison (Figure 16A), significant enrichment was observed in the flavonoid biosynthesis pathway and pathways related to multiple plant secondary metabolite biosynthesis, suggesting that loading treatment activated secondary metabolic systems in *Populus davidiana* × *P. tremuloides* shoot tips. The accumulation of flavonoids, as important antioxidants, may represent a protective strategy against osmotic stress.

In the DLB vs. DLC comparison (Figure 16B), enriched pathways partially overlapped with the DLA group but displayed distinct characteristics. A prominent feature of DLB was the strong enrichment of plant hormone signaling pathways, indicating central regulatory roles of hormonal regulation in physiological responses to osmotic dehydration. Similarly to DLA, enrichment of flavonoid biosynthesis and phenylpropanoid biosynthesis pathways demonstrated that DLB also activated secondary metabolism as a stress-coping mechanism.

### 2.10. Metabolome Analysis

#### 2.10.1. Screening of Differentially Metabolites

**Figure 17:**
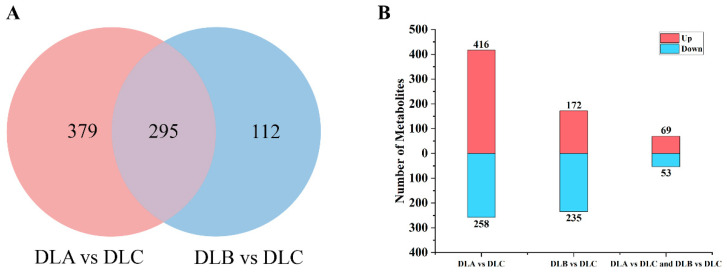
Identification and distribution of differentially accumulated metabolites. (**A**) Venn diagram showing unique and shared metabolites across treatment groups. (**B**) Numbers of upregulated and downregulated metabolites in each comparison. DLA, preculture/loading; DLB, osmotic dehydration; DLC, liquid nitrogen freezing (control).

To investigate the differences in metabolic responses between the two groups, we conducted metabolic profiling analysis on each of them and visualized the results (Figure 17). Through the Venn diagram (Figure 17A), it was found that 674 different metabolites were identified in DLA vs. DLC, among which 416 were upregulated and 258 were downregulated; while in DLB vs. DLC, 407 different metabolites were identified, with 172 upregulated and 235 downregulated. In the DLA vs. DLC experiment, the number of upregulated metabolites was significantly higher than that of downregulated metabolites, suggesting that the physiological state of DLA might be more inclined to activate or accumulate a series of metabolites. In sharp contrast, in the DLB vs. DLC experiment, the number of downregulated metabolites far exceeded that of upregulated metabolites. This result indicates that the treatment of DLB might have dominated a metabolic state characterized by inhibition of metabolite consumption or synthesis.

#### 2.10.2. KEGG Pathway Enrichment Profiling of Metabolomes

KEGG pathway enrichment analysis was performed to elucidate the biological pathways associated with differential metabolites in DLA and DLB comparisons. The results revealed substantial similarity in core metabolic pathway regulation between both treatments. Significantly enriched pathways common to DLA and DLB included Metabolic pathways, Biosynthesis of amino acids, ABC transporters, Carbon metabolism, and 2-Oxocarboxylic acid metabolism. This enrichment pattern indicates that extensive remodeling of primary metabolism—particularly the synthesis and transformation of fundamental biomolecules such as amino acids and organic acids—represents a core metabolic feature shared by both treatments. The enrichment of ABC transporters suggests a critical role of transmembrane transport, potentially reflecting enhanced compartmentalization or excretion of toxic compounds under stress conditions.

**Figure 18:**
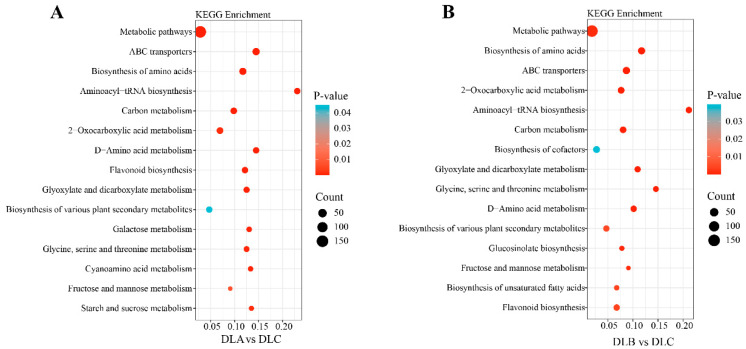
Enrichment Analysis of Differentially Expressed Metabolites in KEGG Pathways. (**A**) KEGG Pathway Enrichment Analysis of DLA and DLC. (**B**) KEGG Pathway Enrichment Analysis of DLB and DLC. DLA, preculture/loading; DLB, osmotic dehydration; DLC, liquid nitrogen freezing (control).

In the DLA vs. DLC comparison (Figure 18A), specific pathway enrichment was observed for Flavonoid biosynthesis and Galactose metabolism. The activation of flavonoid biosynthesis, associated with plant antioxidant responses, aligns with transcriptomic findings indicating that loading treatment preferentially induces stress-adaptive mechanisms.

In contrast, the DLB vs. DLC comparison (Figure 18B) exhibited distinct enrichment in the Biosynthesis of cofactors, Biosynthesis of unsaturated fatty acids, and Glucosinolate biosynthesis. These results suggest that DLB employs a more refined metabolic strategy focused on maintaining redox homeostasis, modulating membrane lipid composition, and mobilizing specialized plant defense systems, reflecting a targeted adaptive response to osmotic dehydration.

### 2.11. Transcription and Metabolome Combined Analysis

#### 2.11.1. Principal Component Analysis

**Figure 19:**
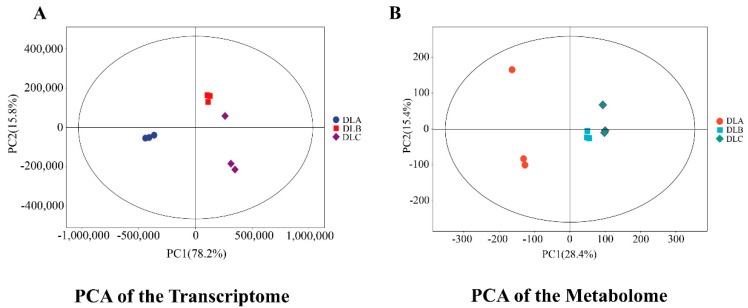
Principal Component Analysis of Transcriptional and Metabolic Profiles. (**A**) PCA of the transcriptome. (**B**) PCA of the metabolome. DLA, preculture/loading; DLB, osmotic dehydration; DLC, liquid nitrogen freezing (control).

To comprehensively evaluate the overall variations and grouping situations of DLA, DLB and DLC samples at the transcriptomic and metabolomic levels, we conducted principal component analysis (PCA) on the gene expression data and metabolite profile data, respectively. The results are shown in Figure 19.

The PCA of the transcriptome (Figure 19A) revealed that the variance contribution rates of the first principal component (PC1) and the second principal component (PC2) were 78.2% and 15.8%, respectively, and they collectively explained up to 94.0% of the total variation. Such a high degree of explanation indicates that PC1 and PC2 can adequately represent the overall characteristics of the dataset. More importantly, the three groups of samples, DLA, DLB, and DLC, showed clear and distinct clustering in the two-dimensional space. The PCA analysis of the metabolome (Figure 19B) showed that the variance contribution rates of PC1 and PC2 were 28.4% and 15.4% respectively, and the cumulative explanation rate (43.8%) was lower than that of the transcriptome. This reflects the complexity of the metabolite spectrum, with its variation driven by more dimensions. Nevertheless, we still observed a clear trend of sample separation. DLC was clearly distinguished from DLA and DLB along the PC1 axis, indicating that both treatments significantly altered the overall metabolite composition. Notably, although the separation degree between DLB and DLA in spatial distribution was not as obvious as that in the transcriptome, it could still be effectively distinguished. This indicates that although DLA and DLB caused different metabolic changes in direction, there might be overlap or intermediate states in some core metabolites, which is consistent with the results we observed in the differential metabolite Venn diagram.

#### 2.11.2. KEGG Analysis for Jointly Regulating the Transcriptome and Metabolome

**Figure 20:**
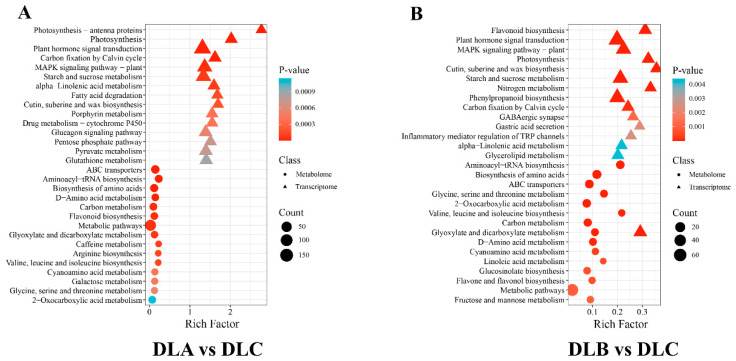
Transcription-Metabolism Combined KEGG Pathway Enrichment Analysis. (**A**) KEGG Pathway Enrichment Analysis of DLA and DLC. (**B**) KEGG Pathway Enrichment Analysis of DLB and DLC. DLA, preculture/loading; DLB, osmotic dehydration; DLC, liquid nitrogen freezing (control).

To systematically clarify the regulatory relationship between gene expression and metabolite accumulation, we conducted a combined KEGG pathway enrichment analysis of transcriptomics and metabolomics for DLA vs. DLC and DLB vs. DLC. This analysis integrated the changes in mRNA and metabolites at two levels, thereby enabling a more comprehensive revelation of the biological pathways that are simultaneously activated or inhibited under specific physiological conditions (Figure 20). The joint analysis revealed significant transcriptional and metabolic co-enrichment in multiple core metabolic and signaling pathways between DLA and DLC (Figure 20A). Firstly, in terms of energy and carbon assimilation, pathways such as Photosynthesis—antenna proteins, Photosynthesis, and Carbon fixation by Calvin cycle were strongly activated at the transcriptome level, while pathways such as Carbon metabolism and Pyruvate metabolism were simultaneously enriched at both the transcriptional and metabolic levels. This indicates that DLA has achieved coordinated enhancement from gene expression to metabolite levels in terms of light energy capture, carbon fixation, and downstream energy metabolism. Secondly, in terms of stress response and secondary metabolism, the MAPK signaling pathway in the transcriptome, glutathione metabolism, and the flavonoid biosynthesis pathway in the metabolome were all significantly enriched, forming a complete response chain from signal perception, antioxidation to defense substance synthesis. At the same time, the pathways of cutin, subcine and wax biosynthesis, and fatty acid degradation were enriched, consistent with the physiological needs of DLA in cell barrier reconstruction and lipid mobilization. This multi-omics co-enrichment pattern clearly depicts that DLA is in a high-energy-consuming and highly stressful physiological state.

Compared with DLA, the combined enriched pathway spectrum of DLB vs. DLC shows different regulatory focuses (Figure 20B). DLB also exhibits activation at the transcriptional level of “photosynthesis” and “Calvin cycle”, but its unique enriched pathways are more focused on the transformation and transportation of substances. For instance, the enrichment of Nitrogen metabolism suggests that DLB adjusts the assimilation and utilization of nitrogen, while the enrichment of Phenylpropanoid biosynthesis and Flavone and flavonoid biosynthesis indicates that the secondary metabolic flow is more specifically directed towards the synthesis of defense compounds. Particularly noteworthy is that Plant hormone signal transduction is significantly enriched in the transcriptome data of DLB, which is consistent with our previous findings in transcriptome analysis, once again emphasizing the core role of hormone-mediated precise regulation in the response mechanism of DLB. At the same time, the continuous enrichment of ABC transporters in the metabolomics data suggests that the transmembrane transport process is crucial for maintaining the metabolic homeostasis of DLB.

By comparing the combined enrichment pathways of DLA and DLB, it can be observed that the transcription and metabolic profiles of DLA jointly point to a pattern of acute stress and energy mobilization. Its regulatory network extensively activates multiple links ranging from signal transduction, photosynthesis to antioxidant and lipid metabolism. The regulation of DLB, on the other hand, appears more restrained and specific. Its core lies in strengthening photosynthesis while precisely regulating nitrogen metabolism, the synthesis and transportation of specific secondary metabolites through hormone signals, in order to achieve physiological balance and adaptation.

#### 2.11.3. Characterization of Key Enzymatic Reactions and Transcriptional Regulation in the Flavonoid Biosynthesis Pathway

**Figure 21:**
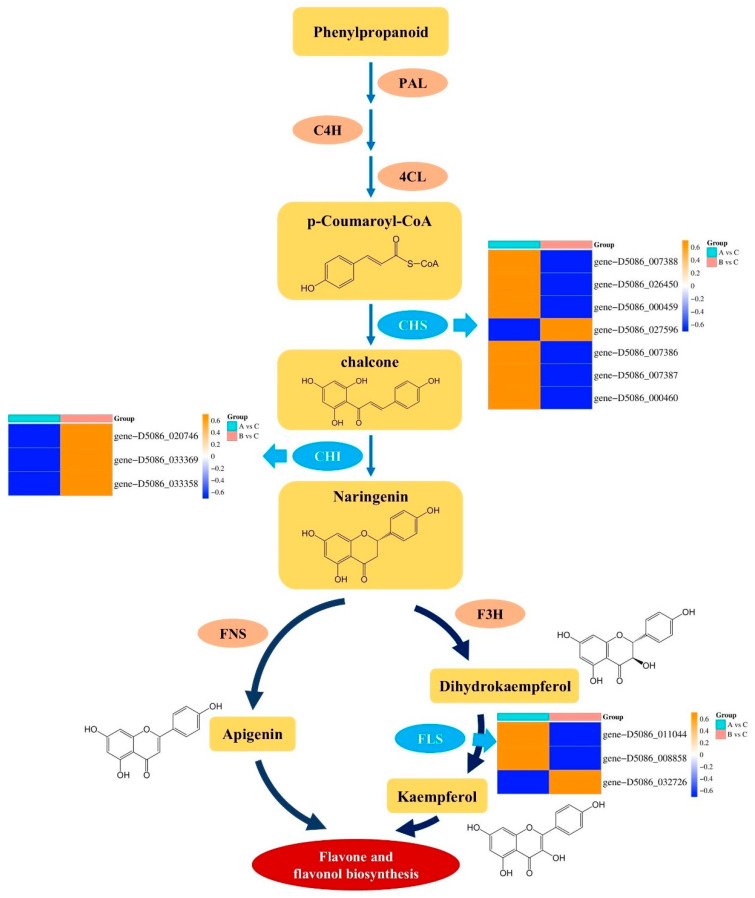
Biosynthetic Pathway of Flavonoids.

Flavonoids serve as crucial secondary metabolites enabling plants to withstand both biotic and abiotic stresses. Elucidating the molecular regulatory mechanisms underlying their biosynthetic pathway is essential for understanding plant stress resistance and secondary metabolic phenotypes [22]. In this study, through integrated transcriptomic and metabolomic analyses, we reconstructed the biosynthetic pathway from phenylpropanoids to flavonoids and flavonols (Figure 21). Furthermore, by correlating the expression patterns of differentially expressed genes, we clarified the functional roles of key enzymatic genes in the regulation of this pathway.

Phenylpropanoids serve as the initial substrates for flavonoid biosynthesis [23]. Through sequential catalysis by phenylalanine ammonia-lyase (PAL), cinnamate 4-hydroxylase (C4H), and 4-coumarate-CoA ligase (4CL), p-coumaroyl-CoA is generated [24]. This segment represents a critical branch of the phenylpropanoid pathway, supplying the direct precursor for flavonoid formation. Both enzymatic activity and gene expression levels of these enzymes fundamentally govern carbon flux allocation into flavonoid synthesis [25]. Under the catalysis of chalcone synthase (CHS), p-coumaroyl-CoA is converted into chalcone, which is subsequently isomerized to naringenin—the core flavonoid precursor [26]. The heatmap depicting CHS-associated genes (right panel) reveals that genes such as *D5086_007388* and *D5086_026450* exhibit significant differential expression in both DLA vs. DLC and DLB vs. DLC comparisons (expression change range: −0.6 to 0.6), indicating that transcriptional regulation of these CHS genes may directly modulate chalcone synthesis efficiency. Similarly, differential expression patterns of chalcone isomerase (CHI)-related genes (left panel, e.g., *D5086_020746* and *D5086_033369*) further demonstrate that heterogeneity in CHI gene expression likely regulates the conversion of chalcone to naringenin, thereby influencing downstream flavonoid branching pathways.

Naringenin serves as a metabolic branch point leading to distinct flavonoid classes through two enzymatic routes [27]: (1) catalysis by flavone synthase (FNS) yielding apigenin (flavones); (2) conversion into dihydrokaempferol by flavanone 3-hydroxylase (F3H), followed by formation of kaempferol (flavonols) via flavonol synthase (FLS). The heatmap of FLS-associated genes (e.g., *D5086_011044*, *D5086_008858*) reveals distinct expression patterns across experimental groups, indicating that transcriptional regulation of FLS plays a critical role in flavonol biosynthesis. In contrast, FNS activity determines the production efficiency of flavones such as luteolin. This differential regulation at the branch point may shift the metabolic flux between flavones and flavonols, ultimately influencing stress-related phenotypes—including antioxidant capacity and UV protection—in plants [28].

## 3. Discussion

Cryopreservation serves as a critical method for the long-term and secure preservation of germplasm resources. It effectively maintains genetic stability and has been widely applied to various materials, including seeds and shoot tips. Furthermore, it demonstrates broad prospects in fields such as the conservation of transgenic materials, virus-free seedling production, and ecological restoration [29]. The success of cryopreservation hinges on whether plant materials can survive and recover after undergoing a series of intense stresses (sudden osmotic changes, dehydration, cold shock). Multi-omics data from this study clearly indicate that shoot-tip cells do not passively endure damage but instead actively initiate a multi-layered, highly coordinated adaptive program. This program begins with the rapid perception and transduction of stress signals. At the DLA stage, transcriptomic data show significant enrichment of the “MAPK signaling pathway—plant” pathway. This pathway serves as a central hub for converting diverse environmental stimuli into intracellular signals in plants [30] and has been demonstrated in species like Arabidopsis to be involved in responses to cold, drought, salt, and oxidative stress [31,32,33]. Its early activation indicates that the mild stress induced by DLA treatment is perceived by the cells, triggering downstream reprogramming of gene expression. At the DLB stage, the signaling network becomes more complex, with one of the most prominent features being the strong enrichment of the “Plant hormone signal transduction” pathway in the transcriptome. The data suggest that when confronted with stress induced by PVS2/PVS3 solutions, shoot-tip cells may precisely coordinate physiological responses by integrating multiple hormone signals [31,34]. This hormone-mediated precise regulation, differing from the more “generalized” MAPK signal initiation seen at the DLA stage, likely represents an optimized strategy for coping with extreme and sustained stress.

Driven by this signaling network, cells activate a series of programs to maintain internal homeostasis. Among these, maintaining redox balance is crucial. Physiological data from this study confirm the occurrence of oxidative stress (increases in MDA and H_2_O_2_), while multi-omics data reveal the comprehensive mobilization of the antioxidant defense system. This includes not only enzymatic systems like SOD, POD, and CAT but also non-enzymatic systems. Notably, the “Glutathione metabolism” pathway is enriched in the transcriptome at both DLA and DLB stages. Glutathione (GSH), as a core antioxidant and redox buffer, plays a key role in oxidative stress tolerance in Arabidopsis [31,35]. Its upregulation, occurring alongside the activation of the flavonoid biosynthesis pathway, helps build a multi-layered ROS scavenging system. Beyond countering oxidative damage, cells must directly address dehydration and cold challenges. Physiological data show that cold acclimation significantly increased the content of soluble sugars, soluble proteins, and free proline, consistent with reports of enhanced cold hardiness in many woody plants [36]. Transcriptomic data further support this adaptive process at the gene expression level, with the activation of pathways such as “Starch and sucrose metabolism” and “Amino acid biosynthesis.” The accumulation of these compatible solutes directly stabilizes cellular structures [37,38]. Additionally, the remodeling of energy metabolism is evident. The co-enrichment of pathways like “Carbon fixation” and “Carbon metabolism” at the DLA stage suggests that cells may maintain or enhance basic energy production to supply raw materials and energy for subsequent high-energy-demanding stress responses, such as the synthesis of large quantities of defensive compounds [39]. The enrichment of “Nitrogen metabolism” at the DLB stage indicates an adjustment in nitrogen assimilation and recycling under severe dehydration to support the synthesis of essential substances.

This study observes significant activation of the flavonoid biosynthesis pathway, evidenced by multi-layered data from transcription (upregulation of key genes like PAL, CHS, FLS) to metabolism (accumulation of flavonols like quercetin). The potent antioxidant activity of flavonoids allows them to effectively quench excess ROS and protect cellular structures [40]. However, when viewed in the context of broader data, flavonoid accumulation should not be seen as an isolated event. Firstly, it is an output branch of the larger “Phenylpropanoid biosynthesis” network [41,42], whose upstream activation provides common precursors for various compounds, including flavonoids and lignin [25,39]. Secondly, flavonoid synthesis is precisely regulated by upstream signals. Its co-enrichment with MAPK and glutathione metabolism at the DLA stage resembles a rapid response under a “stress alarm,” while at the DLB stage, its synthesis is integrated into a more finely tuned network centered on “Plant hormone signal transduction.” In Arabidopsis, hormones like JA and ABA have been shown to specifically regulate different flavonoid branches [43,44,45]. This may explain why the metabolic profile at DLB is more focused compared to DLA—hormonal signals may direct carbon flux toward the most urgently needed specific flavonoid subtypes or other phenylpropanoids at that time [46,47].

Furthermore, metabolomic data hint at other potential protective mechanisms. For instance, the upregulation of genes related to “Cutin, suberin, and wax biosynthesis” may be associated with the reinforcement of the cell wall and outer layers of the plasma membrane to reduce water loss [48]. The consistent enrichment of “ABC transporters” may be involved in expelling harmful substances from cells or locating protective compounds to specific sites, which is crucial for maintaining cytoplasmic homeostasis [49].

In summary, at the DLA stage, cells perceive the initial stress, sound an alarm via pathways like MAPK, rapidly enhance basic metabolism and antioxidant capacity, and initiate the large-scale synthesis of defensive substances, including flavonoids, trading “resource consumption” for immediate protection in preparation for impending severe stress [50]. At the DLB stage, facing intense dehydration, cells activate a precise regulatory network centered on hormonal signaling [51]. The focus of the response shifts toward a more refined “balance-reconstruction,” including the hormone-signal-mediated fine-tuning of specific metabolic pathways, adjustments to primary metabolism like nitrogen metabolism, and enhanced transmembrane transport of substances to maintain homeostasis [52,53].

## 4. Materials and Methods

### 4.1. Plant Material

The *Populus davidiana* × *P. tremuloides* Z9-1 mountain ash is a clonal variety bred through crossbreeding between a superior tree of mountain ash from Weihe Mountain in Heilongjiang Province and an excellent clonal variety of American mountain ash. It is based on its tissue culture seedlings, which are induced to form sterile seedlings using differentiation medium, then transferred to the rooting medium to induce rooting. The propagation cycle is 4 weeks. The temperature in the cultivation room is 25 ± 2 °C, and the photosynthetic effective radiation is 50 μmol·m^−2^·s^−1^, with 16 h of light per day. The formulation of the culture medium and reagents used in the experiment is shown in Table 3, with a pH of 5.8.

### 4.2. Identification of Critical Parameters in the Cryopreservation System for Shoot Tips of Populus davidiana × P. tremuloides

Experimental optimization of cryopreservation protocols was performed using a one-factor-at-a-time approach. Each experimental group comprised 10–15 shoot tips, with three biological replicates per treatment.

The optimized vitrification-based cryopreservation protocol for *Populus davidiana* × *P. tremuloides* shoot tips was conducted as follows: shoot tips (approx. 2.0 mm in length) were excised and precultured on MS medium (pH 5.8) supplemented with 0.5 mol/L sucrose at 4 °C for 3 days. Subsequently, samples were loaded at room temperature for 20 min, treated with PVS3 solution for 60 min, and immersed in liquid nitrogen for 1 h. For recovery, shoot tips were thawed in a 40 °C water bath for 1 min, rinsed twice with loading solution for 20 min, transferred to regeneration medium, and maintained in darkness for 7 days before being returned to standard light conditions.

For the encapsulation–vitrification method, an additional encapsulation step was introduced following preculture: shoot tips were suspended in sodium alginate solution and cross-linked by dripping with CaCl_2_ solution for 20 min under static conditions to form encapsulation beads. The beads were then subjected to loading at room temperature for 20 min, treated with PVS2 solution on ice for 60 min, and cryopreserved in liquid nitrogen for 1 h. Thawing was performed in a 40 °C water bath for 3 min. After unloading, shoot tips were transferred to recovery medium and maintained in darkness for 7 days before being returned to normal light conditions.

Building upon the two cryopreservation protocols described above, a series of single-factor experiments were conducted to systematically optimize the preservation procedure. The screening methodology was adapted from established protocols by Ma [54]. (1) Cold acclimation duration: Second-generation sterile plantlets were cold-acclimated at 4 °C for 0, 1, 2, 3, and 4 weeks. Non-acclimated seedlings served as the control to assess the effect of acclimation period on survival rate. (2) Sucrose preculture concentration: Shoot tips from 2-week cold-acclimated plantlets were inoculated onto MS medium (pH 5.8) supplemented with 0.1, 0.3, 0.5, 0.7, or 0.9 mol/L sucrose and precultured at 4 °C for 3 days. Survival rates were compared among concentrations. (3) Sucrose preculture duration: Shoot tips from 2-week cold-acclimated plantlets were precultured in MS medium with 0.5 mol/L sucrose at 4 °C for 1, 3, 5, 7, or 9 days to evaluate the influence of preculture time on survival. (4) Vitrification solution exposure time: For the vitrification method, shoot tips from 2-week cold-acclimated plantlets were treated with PVS3 for 0, 30, 60, 90, or 120 min. For the encapsulation–vitrification method, samples were treated with PVS2 on ice for 0, 60, 120, 180, or 240 min. Effects of dehydration duration on survival were compared accordingly.

### 4.3. Assessment of Post-Cryopreservation Shoot Tip Survival and Regeneration

Determination of Survival Rate in Cryopreserved Shoot Tips via TTC Staining [54]: The treated shoot tips were immersed in a 2,3,5-triphenyltetrazolium chloride (TTC) solution at a concentration of 4 g/L and incubated in a 37 °C water bath in darkness for 30 min. After staining, the samples were examined under a stereomicroscope. Shoot tips exhibiting red coloration in the apical meristem were recorded as viable, while those without coloration were considered non-viable. The survival rate was calculated as the percentage of viable samples relative to the total number of treated samples.

The regeneration ability assessment was carried out by removing the cells, inoculating the stem tips onto the recovery medium, and then culturing in the dark for 7 days. After that, the medium was switched to normal light conditions. The regeneration was considered successful when the stem tips turned green and new leaves emerged. The regeneration rate was calculated as the percentage of regenerated stem tips out of the total number of inoculated stem tips.

### 4.4. Histological Analysis of Shoot Tip Cell Survival Following Cryopreservation

Histological examination was performed on shoot tips of *Populus davidiana* × *P. tremuloides* following cryopreservation. Fresh, untreated shoot tips served as the positive control, whereas those directly plunged into liquid nitrogen without cryoprotection were designated as the negative control. The experimental groups comprised shoot tips that had undergone a 3-day recovery culture after treatment with the two cryopreservation protocols. The samples were immersed in formalin-acetic acid-alcohol fixative (FAA, 70%) for 30 min, followed by stepwise dehydration in a graded ethanol series (50%, 70%, 80%, 85%, and 95% anhydrous ethanol) with each step lasting 1 h at low temperature. After dehydration, the samples were rinsed twice in xylene (1.5 h each). Subsequently, the samples were infiltrated with paraffin and stored at 4 °C overnight; this infiltration process continued for 5 days, with the paraffin solution being replaced twice daily. The samples were then embedded and solidified in embedding cassettes on a cooling stage. The paraffin-embedded blocks were sectioned into slices of 10–14 μm thickness using a rotary microtome (Leica, RM2245), stained with 0.1% toluidine blue, and finally examined and photographed under a microscope for histological observation [55].

### 4.5. Assessment of Genetic Stability

Genetic Stability Analysis Using ISSR Markers: Tissue-cultured plantlets prior to cryopreservation treatment were used as the control. Genomic DNA was extracted from leaves of the tissue-cultured plantlets using the cetyltrimethylammonium bromide (CTAB) method. Eight primers were selected from an initial set of 100 through preliminary screening for detection. Polymerase chain reaction (PCR) amplification was performed on the samples. The reaction mixture (30 µL total volume) contained 5 µL of 2× Rapid Taq Master Mix, 2 µL of template DNA, 2 µL of primer, and 21 µL of ultrapure water. The PCR protocol was set as follows: initial denaturation at 95 °C for 3 min, 35 cycles of denaturation at 95 °C for 15 s, primer annealing for 15 s, and extension at 72 °C for 1 min, followed by a final extension at 72 °C for 5 min. The amplification products were separated by agarose gel electrophoresis and visualized using a gel imaging system, and clearly distinguishable bands were counted for analysis.

### 4.6. Analysis of Physiological and Biochemical Parameters in Populus davidiana × P. tremuloides Shoot Tips

Shoot tips from *Populus davidiana* × *P. tremuloides* were subjected to various cryo-preservation procedures via the vitrification method and used as experimental material. According to the method of Zhu [56], the changes in soluble sugar and soluble protein contents in *Populus davidiana* × *P. tremuloides* were determined. Reagents from Suzhou Greis Biotechnology Co., Ltd. were used to separately measure the contents of malondialdehyde (MDA, G0109W), hydrogen peroxide (H_2_O_2_, G0112W), catalase (CAT, G0105W), peroxidase (POD, G0107W), superoxide dismutase (SOD, G0101W), ascorbic acid (ASA, G0201W), reduced glutathione (GSH, G0206W), the mass fraction of free proline, and the scavenging ability of superoxide anion and hydroxyl radical.

### 4.7. Detection of Relative Cell Survival Rate During Cryopreservation Process

The relative survival rate of cells was detected by following Wang’s [57] method: Materials were immersed in TTC staining solution and incubated overnight at 28 °C. The following day, samples were rinsed three times with distilled water and transferred into 95% anhydrous ethanol. The ethanol was boiled to extract the TTC-stained products until the shoot tips turned white. Subsequently, the volume was adjusted to 5 mL with 95% anhydrous ethanol. Absorbance was measured at 485 nm using a spectrophotometer. Untreated shoot tips served as the control. The ratio of absorbance of treated shoot tips to that of control shoot tips was taken as the proportion of viable cells.

### 4.8. Integrated Transcriptome and Metabolome Analysis of Populus davidiana × P. tremuloides Shoot Tips During Cryopreservation

#### 4.8.1. Transcriptome Measurement

Following three different types of treatments (DLA, DLB and DLC), RNA was extracted from the shoot tips of poplars (*Populus davidiana* × *P. tremuloides*). Each sample had three biological replicates, and then nine cDNA libraries were constructed [58]. Total RNA was extracted from the shoot tip samples using the conventional CTAB method (Cyltrimethylammonium Bromide), as previously described [59]. The purity and concentration of RNA samples were determined using the Qubit Fluorometer and the Agilent 5400 Bioanalyzer to ensure their suitability for cDNA library construction. The qualified RNA was then used to construct cDNA libraries following the manufacturer’s instructions for the Illumina RNA-Seq Library Preparation Kit (Illumina, CA, USA), and the resulting libraries were subsequently sequenced. Qualified libraries were normalized based on effective concentration and combined proportionally to meet the desired sequencing depth. The final pool was subjected to PE150 (paired-end 150 bp) sequencing on an Illumina No-vaSeq system.

RNA-Seq data processing and quantification were performed using the RSEM (RNA-Seq by Expectation-Maximization) pipeline, which applies an expectation-maximization algorithm to accurately estimate transcript abundance. The reference genome sequence of Populus alba was retrieved from the NCBI database (https://www.ncbi.nlm.nih.gov, accessed on 14 February 2025), a comprehensive platform for plant genomic resources. Following quality control with fastp [60] to remove low-quality reads, the clean reads from each sample were subsequently aligned to the Populus alba reference genome. For comparative analysis, sequencing reads were aligned to the reference genome using STAR [61], followed by quantification of gene expression levels with RSEM. Differential expression analysis was performed on the different poplar samples using edge R 3.36.0 [62]. The significant *p*-values were adjusted for multiple testing using the BH (Benjamini–Hochberg) method. Genes with a false discovery rate (FDR) < 0.05 and an absolute log_2_(fold change) ≥ 1 were considered differentially expressed.

To perform functional enrichment analysis on the identified differentially expressed genes (DEGs), we used the ClusterProfiler software (Version 4.5) for both Gene Ontology (GO) and KEGG pathway analyses. Significant terms were defined as those with a BH adjusted *p*-value < 0.05. Additionally, enrichment analysis was corroborated using the DEGSeq R package (version 1.12.0) and Goseq (v1.46.0) packages under the same significance threshold.

#### 4.8.2. Metabolome Measurement

The stem tips treated with liquid nitrogen were used as the control group (DLC), while the stem tips treated with preculture/loading (DLA) and osmotic dehydration (DLB) were used as the experimental groups. Each group had 3 replicates. Metabolite Extraction (the reagents and instruments used are listed in Table 4 and Table 5): (1) The samples were freeze-dried and ground into powder (60 Hz, 30 s). (2) Approximately 25 ± 1 mg of the lyophilized powder was accurately weighed into a centrifuge tube at low temperature, followed by addition of homogenization beads and 1000 μL of extraction solvent (methanol: acetonitrile: water = 2:2:1, *v*/*v*/*v*) containing isotope-labeled internal standards. (3) The mixture was vortexed for 30 s. (4) Homogenization was performed using a homogenizer (35 Hz, 4 min), followed by ultrasound treatment in an ice-water bath for 5 min. This cycle was repeated three times. (5) The samples were kept at −40 °C for 1 h. (6) Centrifugation was carried out at 4 °C and 12,000 rpm (centrifugal force 13,800× *g*, radius 8.6 cm) for 15 min. (7) The supernatant was filtered through a 0.22 μm microporous membrane, and the filtrate was collected for subsequent instrumental analysis.

Instrumental Analysis: Chromatographic separation was performed on a Vanquish ultra-high-performance liquid chromatography system (Thermo Fisher Scientific, 1290 Infinity II, USA) using a Phenomenex Kinetex C18 column (2.1 mm × 50 mm, 2.6 μm). The mobile phase consisted of (A) water containing 0.01% acetic acid and (B) isopropanol: acetonitrile (1:1, *v*/*v*). The sample tray temperature was maintained at 4 °C, and the injection volume was 2 μL. Mass spectrometry analysis was carried out on an Orbitrap Exploris 120 mass spectrometer operated under Xcalibur software (version 4.4, Thermo Fisher Scientific) for both full-scan and MS/MS data acquisition. The key parameters were set as follows: sheath gas flow rate, 50 Arb; auxiliary gas flow rate, 15 Arb; capillary temperature, 320 °C; full MS resolution, 60,000; MS/MS resolution, 15,000; collision energy, stepped NCE 20/30/40; spray voltage, 3.8 kV (positive ion mode) or −3.4 kV (negative ion mode).

The raw data were converted into mzXML format using ProteoWizard software (Version 3). Metabolite identification was subsequently performed with R packages against the BiotreeDB (V3.0, reference standard database) and BT-Plant (V1.1, plant-specific database) libraries [63], followed by visualization analysis.

### 4.9. Data Processing and Analysis

All data were recorded and organized using Excel 2024. One-way ANOVA tests and Pearson correlation analyses were conducted using SPSS 27.0. Graphs were created using Origin 2024Pro software.

## 5. Conclusions

In conclusion, this study, from the perspective of systems biology, demonstrates that the stem tips of *Populus davidiana* × *P. tremuloides* undergo a multi-level and finely regulated adaptive mechanism during the vitrification preservation process, especially when exposed to osmotic dehydration stress. The core of this mechanism involves rapidly transmitting stress information through hormone signaling, activating the antioxidant system to maintain oxidative balance, and massively reconstructing the secondary metabolic network (especially the flavonoid synthesis pathway) to provide efficient chemical protection. These findings not only deepen our understanding of the stress-resistant physiology of woody plant stem tips but also provide theoretical basis and practical directions for optimizing the cryopreservation preservation technology of forest tree germplasm. For example, adding an appropriate amount of flavonoid synthesis precursors in the pretreatment solution may enable “pre-adaptation” of the stem tips, enhancing their stress resistance, and thereby increasing the survival rate and regeneration rate after vitrification. Future research will focus on using gene editing or overexpression techniques to verify the specific functions of key genes (such as PAL, CHS) in improving the cryopreservation tolerance of poplar and other forest tree germplasm.

## Figures and Tables

**Table 1:** Comparative Analysis of Growth and Rooting Characteristics in Cryopreserved Regenerated Plantlets under In Vitro Culture Conditions.

Regenerants Derived From	Root Formation			Shoot Regrowth		
	Rooting (%)	RM	LLR (cm)	PH (cm)	NN	NFOL
In vitro control	100	10.0 ± 0.5 a	5.1 ± 0.3 a	5.47 ± 1.12 a	6.5 ± 0.3 a	6.5 ± 0.5 a
Cryopreservation	100	9.6 ± 0.6 a	4.9 ± 0.5 a	5.28 ± 0.73 a	6.3 ± 0.5 a	6.2 ± 0.3 a

Note: RM is root number; LLR is length of the longest root; PH is plant height; NN is node number; NFOL is number of fully opened leaves. The same letter ‘a’ indicates no significant difference.

**Table 2:** ISSR-Based Assessment of Genetic Stability in Regenerated Plants following Cryopreservation.

Primer Name	Primer Sequence(5′~3′)	Amplified Band Number
UBC815	CTCTCTCTCTCTCTCTG	4
UBC818	CACACACACACACACAG	5
UBC822	TCTCTCTCTCTCTCTCA	7
UBC850	GTGTGTGTGTGTGTGTYC	5
UBC874	CCCTCCCTCCCTCCCT	6
UBC888	BDBCACACACACACACA	8
UBC889	DBDACACACACACACAC	8
UBC890	VHVGTGTGTGTGTGTGT	7
Total number of strips		50

**Table 3:** Medium and reagent formulation used in the test.

Culture Media and Reagents	Recipe
Differentiation medium	MS 6-benzylaminopurine (6-BA) 0.5 mg/L Naphthaleneacetic acid (NAA) 0.05 mg/L Sucrose 20 g/L Agar 7 g/L
Rooting medium	1/2 MS Indole-3-butyric acid (IBA) 0.5 mg/L Naphthaleneacetic acid (NAA) 0.02 mg/L Sucrose 20 g/L Agar 7 g/L
Recovery medium	MS corn medium (ZT) 0.5 mg/L, gibberellic acid (GA3) 1 mg/L, sucrose 20 g/L, 7 g/L
Loading solution	MS 2 M glycerol 0.4 M sucrose
PVS2 solution	MS sucrose 0.4 mol/L, glycerol 30%, ethylene glycol 15%, dimethyl sulfoxide (DMSO) 15%
PVS3 solution	50% sucrose, 50% glycerin
Unloading solution	MS 1.2 M sucrose
Sodium alginate solution	MS 2 mol/L glycerol 2.5% sodium alginate
Calcium chloride solution	MS + 0.1 mol/LCaCl2

**Table 4:** Reagents for Metabolomics Analysis.

Chemical Name	CAS	Purity	Source
Methanol	67-56-1	LC-MS	CNW Technologies (Shanghai Bailey Biotechnology Co., Ltd.)
Acetonitrile	75-05-8	LC-MS	CNW Technologies (Shanghai Bailey Biotechnology Co., Ltd.)
ddH_2_O	-	-	Watsons (Guangzhou, Guangdong Province)
Acetic acid	64-19-7	LC-MS	SIGMA-ALDRICH (Sigma-Aldrich (Shanghai) Trading Co., Ltd.)
2-Propanol	67-63-0	LC-MS	CNW Technologies (Shanghai Bailey Biotechnology Co., Ltd.)

**Table 5:** Instruments for Metabolomics Analysis.

Instrument	Model	Manufacturer
UHPLC	Vanquish	Thermo Fisher Scientific (Massachusetts, USA)
HRMS	Orbitrap Exploris 120	Thermo Fisher Scientific (Massachusetts, USA)
Centrifuge	Heraeus Fresco17	Thermo Fisher Scientific (Massachusetts, USA)
Analytical balance	BSA124S-CW	Sartorius (Göttingen, Germany)
Ultrasonic bath	PS-60AL	Shenzhen Leidebang Electronic Co., Ltd.
Homogenizer	JXFSTPRP-24	Shanghai Jingxin Technology Co., Ltd.
Freeze dryer	LGJ-10C	Sihuan Furuikeyi Technology Development Co., Ltd.

## Data Availability

The original contributions presented in the study are included in the article, further inquiries can be directed to the corresponding authors.

## References

[1] Huang M.B., Han S., Li M.M., Jiao Z.Y., Li Z., Wang Y.J., Liu C., Wang H.L., Yin W., Xia X. (2025). The Cysteine-Rich Peptide PeGASA15 from Populus Euphratica Enhances Drought and Salinity Tolerance in Poplar. Ind. Crops Prod..

[2] Larsen S.U., Hestbjerg H., Jørgensen U., Kongsted A.G. (2024). Ensiling of Willow and Poplar Biomass Is Improved by Ensiling Additives. Agriculture.

[3] Ma X.Y., Blystad D.-R., Wang Q.C., Hamborg Z. (2025). Cryopreservation of Rubus Viruses in Raspberry Shoot Tips via Droplet-Vitrification: Assessment of Viral Preservation, Localization, and Post-Thaw Transmission Capacity. Plant Methods.

[4] Martín C., Nagel M., Ibáñez M.A., Senula A., Pirredda M., González-Benito M.E. (2025). Genetic and Epigenetic Fidelity of Garlic Cryopreserved Plant Material Compared to Field Collections. Sci. Hortic..

[5] Kulus D. (2020). Shoot Tip Cryopreservation of *Lamprocapnos spectabilis* (L.) Fukuhara Using Vitrification, Droplet-Vitrification and Encapsulation-Vitrification Techniques. Cryobiology.

[6] Sakai A., Engelmann F. (2007). Vitrification, Encapsulation-Vitrification and Droplet-Vitrification: A Review. CryoLetters.

[7] Langis R., Steponkus P.L. (1990). Cryopreservation of Rye Protoplasts by Vitrification. Plant Physiol..

[8] Chang Y., Reed B.M. (2001). Preculture Conditions Influence Cold Hardiness and Regrowth of Pyrus Cordata Shoot Tips after Cryopreservation. HortScience.

[9] Engelmann F. (2004). Plant Cryopreservation: Progress and Prospects. In Vitro Cell. Dev. Biol.–Plant.

[10] Matsumoto T., Mochida K., Itamura H., Sakai A. (2001). Cryopreservation of Persimmon (*Diospyros kaki* Thunb.) by Vitrification of Dormant Shoot Tips. Plant Cell Rep..

[11] Towill L.E., Towill L.E., Bajaj Y.P.S. (2002). Cryopreservation of Plant Germplasm: Introduction and Some Observations. Cryopreservation of Plant Germplasm II.

[12] Htwe C.S.S., Rajkumar S., Pathania P., Agrawal A. (2023). Transcriptome Profiling during Sequential Stages of Cryopreservation in Banana (*Musa* AAA Cv Borjahaji) Shoot Meristem. Plants.

[13] Volk G.M., Harris J.L., Rotindo K.E. (2006). Survival of Mint Shoot Tips after Exposure to Cryoprotectant Solution Components. Cryobiology.

[14] Volk G.M., Walters C. (2006). Plant Vitrification Solution 2 Lowers Water Content and Alters Freezing Behavior in Shoot Tips during Cryoprotection. Cryobiology.

[15] Durães S.B.Í., Renato P., dos Reis M.V., Vasconcellos V.B.L., Monteiro M.E., de Campos J.M.S. (2023). Seed Cryopreservation without Vitrification (PVS2) Induces Oxidative Stimuli to Promote Endoreplication in Red Pitaya Seedlings. Plant Cell Tissue Organ Cult..

[16] de Oliveira Prudente O., Paiva R., Domiciano D., de Souza L.B., Carpentier S., Swennen R., Silva L.C., Nery F.C., Máximo W.P.F., Panis B. (2019). The Cryoprotectant PVS2 Plays a Crucial Role in Germinating *Passiflora ligularis* Embryos after Cryopreservation by Influencing the Mobilization of Lipids and the Antioxidant Metabolism. J. Plant Physiol..

[17] Montaña D.C., Serradilla M.J., García M.J.B., Micharet B.V. (2025). Postharvest Melatonin Treatments Reduce Chilling Injury Incidence in the “Angeleno” Plum by Enhancing the Enzymatic Antioxidant System and Endogenous Melatonin Accumulation. Sci. Hortic..

[18] Ayyub S., Khan A.S., Anwar R., Rehman A., Shah H.M.S., Ali S. (2025). Kojic Acid Suppresses Browning Incidence, Maintains Quality and Sensory Attributes of Harvested Loquat Fruit by Upregulating Enzymatic and Non-Enzymatic Antioxidants. PostHarvest Biol. Technol..

[19] Xu S., Wei J., Wang X., Zhang R., Gao J., Li X., Wang C., Ye Y. (2025). Phenolic Compounds Enhance Aluminum Tolerance in Chinese Fir (*Cunninghamia lanceolata*) by Regulating Reactive Oxygen Species Homeostasis and Cell Wall Properties Under Aluminum Stress. Plants.

[20] Alharbi K., Desoky E.S.M., Almuziny M., Abuzaid A.O., Elsaoud A.M.A., Algopishi U.B., Serag A.M., Saadony M.T.E., Mathew B.T., Tarabily K.A.E. (2025). Foliar-Applied Selenium Nanoparticles Improve Antioxidant Defense and Photosynthetic Efficiency to Enhance Salt Stress Tolerance in Cowpea (*Vigna unguiculata* L.). Sci. Hortic..

[21] KS A., Puthur J.T., Dhankher O.P. (2025). Plant Cell Wall Remodeling under Toxic Metal Stress: Structural Adaptation and Functional Implications. Environ. Sci. Technol..

[22] Ying X., Cui L., Peng L., Yuepeng S. (2023). Interactions between PtoYABBY5 and PtoMYB43 Regulate Flavonoid Synthesis in Populus Tomentosa. Ind. Crops Prod..

[23] Jiyoung R., Jiyoung S., Youngshim L., Yoongho L., Joong-Hoon A., Hor-Gil H. (2005). Identification of Syn- and Anti-Anethole-2,3-Epoxides in the Metabolism of Trans-Anethole by the Newly Isolated Bacterium *Pseudomonas putida* JYR-1. J. Agric. Food Chem..

[24] Maham N., Chamani E., Mohebodini M., Tariverdizadeh N. (2025). Optimizing Biosynthesis: The Role of LED Light Spectra in Regulating Phenolic and Flavonoid Accumulation in *Matricaria chamomilla* L. Root Cultures. BMC Plant Biol..

[25] Yuan X., Shuai L., Deng X., Yang X., Zhou Y., Jiang Y., Wang B. (2026). Exogenous MeJA Pretreatment Induces Specific Flavonoid Biosynthesis and Glycosylation to Alleviate Chilling Injury in Cold-Stored Cucumber Fruit. Postharvest Biol. Technol..

[26] Li S., Yang K., Feng D., Zhang Y. (2025). Enhanced Antioxidant Properties and Bioavailability of Naringenin through EGCG-Modified Alfalfa Ferritin Nanoparticles. J. Agric. Food Res..

[27] Park N., Ham Y., Cha S., Jang B., Kim G., Baek S.H., Hahn J.S. (2025). Metabolic Engineering of *Yarrowia lipolytica* for Enhanced Production of Naringenin-Derived Flavonoids: Apigenin and Acacetin. J. Agric. Food Chem..

[28] Xueying M., Wei Y., Oskar L., Merja N., Heikki K., Baoru Y. (2017). Role of Flavonols and Proanthocyanidins in the Sensory Quality of Sea Buckthorn (*Hippophaë rhamnoides* L.) Berries. J. Agric. Food Chem..

[29] Roque-Borda C.A., Kulus D., Vacaro De Souza A., Kaviani B., Vicente E.F. (2021). Cryopreservation of Agronomic Plant Germplasm Using Vitrification-Based Methods: An Overview of Selected Case Studies. Int. J. Mol. Sci..

[30] Zhou H., Xie Y., Jiang Y., Nadeem H., Wang Y., Yang N., Zhu H., Tang C. (2023). GhTLP1, a Thaumatin-like Protein 1, Improves Verticillium Wilt Resistance in Cotton via JA, ABA and MAPK Signaling Pathway-Plant Pathways. Int. J. Biol. Macromol..

[31] Ren L., Zhang D., Jiang X.-N., Gai Y., Wang W.-M., Reed B.M., Shen X.-H. (2013). Peroxidation Due to Cryoprotectant Treatment Is a Vital Factor for Cell Survival in *Arabidopsis cryopreservation*. Plant Sci..

[32] Namba J., Harada M., Shibata R., Toda Y., Maruta T., Ishikawa T., Shigeoka S., Yoshimura K., Ogawa T. (2024). AtDREB2G Is Involved in the Regulation of Riboflavin Biosynthesis in Response to Low-Temperature Stress and Abscisic Acid Treatment in *Arabidopsis thaliana*. Plant Sci..

[33] Zhang H., Chen J., Wang W., Li Q., Zhang W., Li B., Zhang X., Hu J., Wang Z., Liu Y. (2025). Integrated Transcriptomic and Metabolomic Analysis Reveals the Molecular Mechanisms of Cadmium Stress Response in Rapeseed (*Brassica napus* L.): Insights into Glutathione and Sulfur Metabolism Pathways. Ind. Crops Prod..

[34] Liu D., Jiao Q., Liu H., Fan L., Yu P., Jiang G., Chen Y., Agathokleous E., Jie X., Liu S. (2025). Wheat Roots Adapt to Potassium Deficiency through Coordinated Antioxidant, Hormonal, and Metabolic Reprogramming: Insight from Morpho-Physiological, Biochemical, and Transcriptomic Analysis. Plant Stress.

[35] Gao B., Tang W., Danilov D.A., Han P., Hua J., Xu Y., Feng Z., Kryukovskiy A., Wu J., Wang J. (2025). Overexpression of the Glutathione Synthase Gene PsGSH2 Enhances Cadmium Stress Tolerance in Transgenic *Arabidopsis thaliana*. Plant Cell Rep..

[36] Wang Y.-M., Zhang Y.-M., Zhang X., Zhao X., Zhang Y., Wang C., Wang Y.-C., Wang L.-Q. (2021). Poplar PsnICE1 Enhances Cold Tolerance by Binding to Different Cis-Acting Elements to Improve Reactive Oxygen Species-Scavenging Capability. Tree Physiol..

[37] Liu C., Xue Y., Guo J., Ren H., Jiang S., Li D., Song J., Zhang Z. (2021). Citric Acid and Sucrose Pretreatment Improves the Crispness of Puffed Peach Chips by Regulating Cell Structure and Mechanical Properties. LWT.

[38] Mauro M.A., Dellarosa N., Tylewicz U., Tappi S., Laghi L., Rocculi P., Rosa M.D. (2016). Calcium and Ascorbic Acid Affect Cellular Structure and Water Mobility in Apple Tissue during Osmotic Dehydration in Sucrose Solutions. Food Chem..

[39] Zhu W., Wu H., Yang C., Shi B., Zheng B., Ma X., Zhou K., Qian M. (2023). Postharvest Light-Induced Flavonoids Accumulation in Mango (*Mangifera indica* L.) Peel Is Associated with the up-Regulation of Flavonoids-Related and Light Signal Pathway Genes. Front. Plant Sci..

[40] Williamson G., Kay C.D., Crozier A. (2018). The Bioavailability, Transport, and Bioactivity of Dietary Flavonoids: A Review from a Historical Perspective. Compr. Rev. Food Sci. Food Saf..

[41] Rai A., Skårn M.N., Elameen A., Tengs T., Amundsen M.R., Bjorå O.S., Haugland L.K., Yakovlev I.A., Brurberg M.B., Thorstensen T. (2025). CRISPR-Cas9-Mediated Deletions of FvMYB46 in Fragaria Vesca Reveal Its Role in Regulation of Fruit Set and Phenylpropanoid Biosynthesis. BMC Plant Biol..

[42] Baldi P., Asquini E., Nicolussi Golo G., Populin F., Moser M. (2023). Isoenzymes of the Flavonoid and Phenylpropanoid Pathways Show Organ-Specific Regulation during Apple Fruit Development. Int. J. Mol. Sci..

[43] Xudong Z., Xing H., Yan L., Faqing T., Qinshi Z., Weiqi L. (2021). Polar Auxin Transport May Be Responsive to Specific Features of Flavonoid Structure. Phytochemistry.

[44] He X., Fang J., Hong B., Zhang X., Li L., He Y., Chen C., Liang S., Xu Z., Peng C. (2025). Research on the Hormonomics of Three Lilium Species and Their Flavonoid Diversification and Specificity. Antioxidants.

[45] Wangshu M., Dongdong L., Zisheng L., Linchun M., Tiejin Y. (2015). Transcriptomic Analysis Reveals Possible Influences of ABA on Secondary Metabolism of Pigments, Flavonoids and Antioxidants in Tomato Fruit during Ripening. PLoS ONE.

[46] Wu P., Yang Z., Kong Q., Cui H., Liu Y., Dong R., Zheng C., Liu H., Cui J. (2025). Phytohormone Response to Exogenous Nitric Oxide in Cucumber Under Low-Temperature Stress. Plants.

[47] Lu Q., Chen S., Shan B., Wei A., Luo Y., Wu L., Jiang Q., Chen Z. (2025). The Ionome–Hormone–Flavonoid Network Shapes Genotype-Dependent Yield Adaptation in Sugarcane. Plants.

[48] Zhang M., Wang J., Liu R., Liu H., Yang H., Zhu Z., Xu R., Wang P., Deng X., Xue S. (2022). CsMYB96 Confers Water Loss Resistance in Citrus Fruit by Simultaneous Regulation of Water Transport and Wax Biosynthesis. J. Exp. Bot..

[49] Wang X., Wang J., Ai W., Zhao N., Zhang Y., Xu L., Kong X., Gao J., Zheng C., Yang B. (2025). Transcriptomic and Metabolomic Analyses Uncover the Molecular Defense Strategy of *Mikania micrantha* under Feeding Stress from *Pachypeltis micranthus*. Plant Stress.

[50] Zhang Q., Ruan J., Mumm R., De Vos R.C.H., Liu M.-Y. (2022). Dynamic Changes in the Antioxidative Defense System in the Tea Plant Reveal the Photoprotection-Mediated Temporal Accumulation of Flavonoids Under Full Sunlight Exposure. Plant Cell Physiol..

[51] Das A., Adak M.K. (2025). Unfolding of Minerals Acquisition in Plants: The Role of Secondary Messengers, Phytohormones and Epigenetics Under Dehydration Stress. J. Plant Growth Regul..

[52] He B., Peng Y., Tong J., Xu D., Dong Y., Zhou Y., Tang Y., Zhang S., Fang L., Mao J. (2025). Transcriptomic and Metabolomic Analyses Reveal Differing Phytohormone Regulation in Rhododendron Cultivars in Response to Azalea Lace Bug (*Stephanitis pyrioides*). Horticulturae.

[53] Ahmed N., Li J., Li Y., Deng L., Deng L., Chachar M., Chachar Z., Chachar S., Hayat F., Raza A. (2025). Symbiotic Synergy: How Arbuscular Mycorrhizal Fungi Enhance Nutrient Uptake, Stress Tolerance, and Soil Health through Molecular Mechanisms and Hormonal Regulation. IMA Fungus.

[54] Ma M., Wang X., Zhang C., Pak S., Wu H., Yang J., Li C. (2023). Enhancing the Cryopreservation System of Larch Embryogenic Culture by Optimizing Pre-Culture, Osmoprotectants, and Rapid Thawing. Forests.

[55] Li Z., Wang Y., Qi Y., Chen S., Liu Y., Fan G., Zhang W., Chen S. (2025). Transcription Factor PsnERF1 Regulates Leaf Morphological Development in Poplar. Plant Cell Tissue Organ Cult..

[56] Zhu L., Zhang C., Yang N., Cao W., Li Y., Peng Y., Wei X., Ma B., Ma F., Ruan Y.-L. (2024). Apple Vacuolar Sugar Transporters Regulated by MdDREB2A Enhance Drought Resistance by Promoting Accumulation of Soluble Sugars and Activating ABA Signaling. Hortic. Res..

[57] Wang B., Li J.-W., Zhang Z.-B., Wang R.-R., Ma Y.-L., Blystad D.-R., Keller E.R.J., Wang Q.-C. (2014). Three Vitrification-Based Cryopreservation Procedures Cause Different Cryo-Injuries to Potato Shoot Tips While All Maintain Genetic Integrity in Regenerants. J. Biotechnol..

[58] Zhang J., Wang D., Chen P., Zhang C., Yao S., Hao Q., Agassin R.H., Ji K. (2023). The Transcriptomic Analysis of the Response of *Pinus massoniana* to Drought Stress and a Functional Study on the ERF1 Transcription Factor. Int. J. Mol. Sci..

[59] Kiss T., Karácsony Z., Gomba-Tóth A., Szabadi K.L., Spitzmüller Z., Hegyi-Kaló J., Cels T., Otto M., Golen R., Hegyi Á.I. (2024). A Modified CTAB Method for the Extraction of High-Quality RNA from Mono-and Dicotyledonous Plants Rich in Secondary Metabolites. Plant Methods.

[60] Chen S., Zhou Y., Chen Y., Gu J. (2018). Fastp: An Ultra-Fast All-in-One FASTQ Preprocessor. Bioinformatics.

[61] Dobin A., Davis C.A., Schlesinger F., Drenkow J., Zaleski C., Jha S., Batut P., Chaisson M., Gingeras T.R. (2013). STAR: Ultrafast Universal RNA-Seq Aligner. Bioinformatics.

[62] Robinson M.D., McCarthy D.J., Smyth G.K. (2010). edgeR: A Bioconductor Package for Differential Expression Analysis of Digital Gene Expression Data. Bioinformatics.

[63] Zhou Z., Luo M., Zhang H., Yin Y., Cai Y., Zhu Z.-J. (2022). Metabolite Annotation from Knowns to Unknowns through Knowledge-Guided Multi-Layer Metabolic Networking. Nat. Commun..

